# Feasibility trial of a self-help digital intervention for functional cognitive disorder

**DOI:** 10.1093/braincomms/fcaf248

**Published:** 2025-06-19

**Authors:** Verónica Cabreira, Lisbeth Frostholm, Jon Stone, Alan Carson

**Affiliations:** Centre for Clinical Brain Sciences, University of Edinburgh, Edinburgh, 49 Little France Crescent, Edinburgh EH16 4SB, UK; Department of Clinical Medicine, Aarhus University, Palle Juul-Jensens Boulevard 118200 Aarhus N, Denmark; Department of Functional Disorders, Aarhus University Hospital, Palle Juul-Jensen Boulevard 11DK-8200 Aarhus N, Denmark; Centre for Clinical Brain Sciences, University of Edinburgh, Edinburgh, 49 Little France Crescent, Edinburgh EH16 4SB, UK; Centre for Clinical Brain Sciences, University of Edinburgh, Edinburgh, 49 Little France Crescent, Edinburgh EH16 4SB, UK

**Keywords:** functional cognitive disorder, internet intervention, mobile app, feasibility, cognitive behavioural therapy

## Abstract

Functional cognitive disorder is the cognitive subtype of functional neurological disorders. Symptoms are associated with a high level of distress and typically arise due to various risk factors including abnormal focused attention, high memory expectations and poor metacognition. Easily accessible specialized treatments for functional cognitive disorder are needed. Digital interventions are less costly and scalable and allow individuals to engage in self-care in a flexible manner. In this study, we aimed to (i) evaluate the feasibility and safety of a novel self-help digital intervention based on principles of cognitive behavioural therapy and related approaches, (ii) explore preliminary effects of this intervention and (iii) learn about participants’ experience with the intervention. Patients were recruited via a neuropsychiatry clinic. They autonomously completed a 6-week intervention, with phone and email technical support, and were assessed at baseline and at the end of the intervention, using self-reported questionnaires. Besides feasibility outcomes, main measures were the metamemory in adulthood questionnaire, subjective memory complaints, clinical global impression of change, Brief Illness Perception Questionnaire, Behavioural Responses to Illness Questionnaire, Compensatory Cognitive Strategies Questionnaire, depressive and anxiety symptoms, work and social functioning and quality of life. A within-group design was employed. Means, standard deviations and effect sizes were estimated. Post-treatment semi-structured interviews were conducted to further explore the acceptability and usability of the programme. Of the 38 patients eligible, 37 completed the baseline questionnaires (97% recruitment rate). Thirty started the intervention (54% female, mean age 50.4). Four dropped out (4/30, 13%), with 23/30 (77%) completing three or more modules, and the attrition rate was 37%. Twenty-seven treatment-related negative effects were reported (5% of total). Overall satisfaction with the programme was high with 62% (16/26) reporting feeling ‘satisfied’ or ‘very satisfied’. Sixteen (62%) reported improvement in their cognitive symptoms (‘minimally improved’, ‘much improved’ and ‘very much improved’). Paired *t*-tests showed significant improvements on illness perceptions (mean change −6.65, *P* = 0.001), depressive symptoms (mean change −2.96, *P* = 0.008) and quality of life (mean change 10.06, *P* = 0.03) at post-treatment. Five patients were dissatisfied with the lack of personalization and found the content unhelpful, with some experiencing increased anxiety. While the intervention met most of its feasibility benchmarks, efficacy needs to be explored in a future randomized controlled trial. Self-help digital therapy may be a flexible and cost-effective option to increase availability and accessibility of specialized treatment for functional cognitive disorder, within a stepped care model, as a complement to other multidisciplinary face-to-face interventions.

## Teaser

Cabreira *et al*. report feasibility and acceptability data on a novel digital self-help intervention for functional cognitive disorder tested in a pilot single-arm trial. The cognitive behavioural therapy–based programme led to symptom improvement in 62% of the participants and potential benefits in illness perceptions, depressive symptoms and quality of life.

## Graphical Abstract

**Graphical Abstract:**
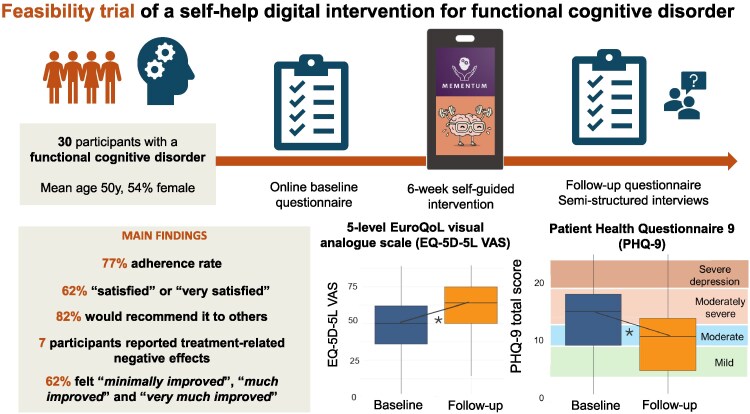

## Introduction

Functional cognitive disorder (FCD) is the cognitive subtype of functional neurological disorders (FNDs).^1^ Patients usually report difficulties remembering conversations, misplacing items or temporarily forgetting names of people, with evidence of marked internal inconsistency in cognitive functioning. These symptoms are associated with elevated levels of distress, disability and work and social impairment, motivating healthcare seeking.^2,3^ Symptoms typically arise due to a variety of risk factors including abnormal focused attention, high expectations and concern about memory, which tends to drive ensuing monitoring and concern that further reduce memory efficiency.^4^ While FCD may present in isolation, it is often part of a broader syndrome such as fibromyalgia or chronic fatigue syndrome.^5,6^ Around 50% of the patients have comorbid depression, anxiety and sleep problems.^3,5,7^

Developing evidence-based interventions for FCD is a clinical priority. Preliminary evidence suggests that psychotherapy focused on modification of memory expectations with cognitive restructuring and stress-reduction techniques is potentially beneficial in FCD, but findings need replication.^8,9^ Other therapist-guided interventions based on cognitive behavioural therapy, acceptance and commitment therapy and cognitive rehabilitation strategies are currently under development.^10-12^ However, challenges including being resource- and time-intensive (5–14 sessions), cost and limited expertise and availability remain. In practice, a UK survey in memory clinics identified that over two-thirds of the clinicians immediately discharge FCD patients to primary care, mostly with simple reassurance.^13^ A recent Delphi consensus highlighted the lack of available treatments and risk of iatrogenic harm and symptom chronicity with exposure to inappropriate therapies.^14^

Digital interventions are less costly and potentially scalable, but they also allow individuals to engage in self-care and receive support in a way that suits their own unique circumstances.^5^ Self-help support has been offered in a range of neurological conditions, including epilepsy,^15^ multiple sclerosis with comorbid cognitive difficulties^16^ and functional disorders,^17-19^ in a more cost-effective manner and with good patient satisfaction. Although still in initial stages comparatively to other internet-delivered interventions, there is some preliminary evidence to support app-based health interventions.^20-24^ Mobile apps have the potential to minimize human contact variables (higher fidelity),^25^ enable integration of different therapeutic approaches and adjustment of the treatment dosage to their tolerance and facilitate the application of therapeutic strategies in daily life, enhancing portability and accessibility.^25^ Yet, digital interventions are often based around a unitary model of a disorder and so offer less flexibility in comparison to traditional face-to-face therapy. In contrast with a dynamic interaction between a therapist and a patient, self-guided interventions assume that the users can effectively navigate a limited set of choices, which may not always facilitate the same level of self-reflection and insight. Despite the vast array of digital interventions for cognitive symptoms available, none specifically tackles the core mechanisms underlying FCD.^26^ To address this challenge, we developed Mementum—a novel digital self-help intervention for FCD, optimized according to both theory-driven therapeutic methods and users’ feedback.

We hypothesized that self-help with automatic guidance delivered via a mobile app is a feasible way of delivering an educational and potentially therapeutic intervention for patients with FCD. The aims of this pilot study are to (i) evaluate the feasibility and safety of a self-help digital intervention, (ii) explore preliminary effects of the novel intervention and (iii) learn about participants’ experience with the study and the intervention to inform a future randomized controlled trial (RCT).

## Materials and methods

### Study design

This is a single-arm trial assessing the feasibility, safety and preliminary efficacy of the novel intervention and trial process including recruitment and measurement protocol.

Reporting follows the CONSORT extension to pilot and feasibility trials,^27^ with adaptations to the non-randomized single-arm design. The study protocol was registered in OSF (https://osf.io/u72pv/) and received ethical approval from North of Scotland Research Ethics Service REC reference 23/NS/0102.

Recruitment was from a neuropsychiatry outpatient clinic in NHS Lothian, Scotland, UK, between December 2023 and September 2024. Patients could be referred by primary care, Neurology and Neuropsychiatry services in NHS Lothian and NHS Fife, for eligibility and recruitment assessments. Eligible patients received a leaflet about the study procedures and could sign up for the study in the clinic or online. Patients provided in person or digital written consent.

### Participants

To be included, participants had to have an established diagnosis of FCD according to the definition proposed by Ball *et al.*,^1^ be 18 years or older, have capacity to provide written informed consent, access to a smartphone/tablet with internet and sufficient English proficiency. Patients were excluded if they had disabling cognitive symptoms fully explained by a primary psychiatric disorder (e.g. bipolar affective disorder or schizophrenia), a diagnosis of probable Alzheimer’s type dementia or another dementia syndrome, an intellectual disability interfering with the ability to comply with the study protocol and give informed consent and another functional disorder dominating the clinical picture or were taking part in other ongoing intervention testing study related to their cognitive symptoms.

### Intervention

Mementum was designed as a self-help app supporting the delivery of education about predisposing, precipitating and maintaining factors contributing to functional cognitive symptoms. Its development is described separately.^28^ It includes (i) CBT-based strategies aimed at shifting how participants think and feel about their memory and encouraging behavioural activation to reduce reliance on excessive safety strategies and promote tolerance to cognitive failures, (ii) mindfulness-based strategies to decrease hyperarousal and cognitive symptom-related distress (iii) cognitive rehabilitation strategies to manage external and internal distractions and increase ‘memory successes’ (e.g. spaced retrieval, active noticing) and (iv) optional content on symptoms that contribute to a depletion of attentional resources reinforcing inattentive cognitive lapses (e.g. pain, fatigue, poor sleep). The programme was delivered over 6 weeks. This duration was selected based on similar self-help interventions and evidence suggesting that longer treatment duration does not necessarily translate to higher treatment effects.^26,28^

The content was distributed across different interactive features: (i) seven content modules including text, visuals and videos; (ii) homework tasks to reinforce learning including metacognitive strategies, behavioural activation and establishment of goals to increase valued activities; (iii) a library of brief exercises including breathing, attention training, deep muscle relaxation, grounding and mindful walking that may improve memory performance^29,30^ and enhance meta-awareness,^31^ (iv) a memory diary for self-assessment and promotion and memory efficacy rated on a Likert scale from 1 to 5; (v) a symptom check-in that enabled users to receive tailored recommendations for their needs (e.g. relevant modules, tasks and exercises); (vi) patient stories presented in text and audio formats; (vii) a library of external resources; and (viii) FAQs.^28^

**Figure 1:**
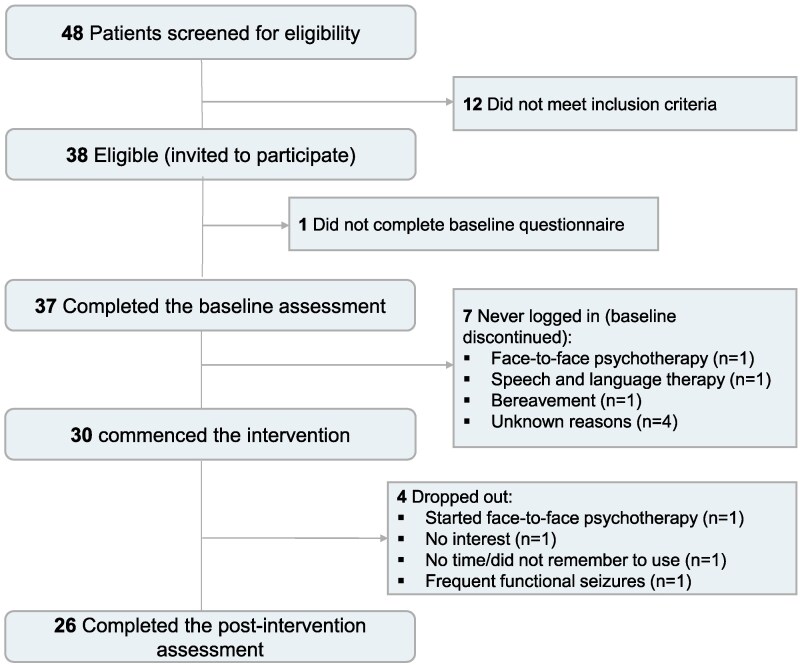
Participant flow diagram.

Patients downloaded the mobile app onto their smartphones and were instructed to use the app at their own pace and preference, aiming to complete a module and a homework task per week, equivalent to 1–2 h per week. Regular emails and bi-weekly phone calls to improve engagement and provide technical support were offered by the team. Additionally, push notifications were sent once a week alongside automatic emails after completing each module to promote adherence. Patients had unrestricted access to usual care during the study and were offered a follow-up appointment post-intervention. Both the platform and treatment content were developed *de novo* following the Medical Research Council guidance on developing and evaluating complex interventions.^32^ At the time of the study, the app was free to participants and access was password protected; each participant received a voucher at the time of enrolment^28^ (Supplementary Tables 1 and 2 and Fig. 1 provide further details on the intervention content and design features).

### Measurements

Baseline data (T0) was collected after consent signing. All participants received automated email reminders (up to three) to complete the follow-up questionnaire at 6 weeks (T1) and were consecutively contacted for a semi-structured interview after completing the intervention, if they were available. The Cogniss® platform, which hosts the app, automatically recorded data on user engagement with the intervention, namely, access to modules, symptom monitoring, tasks and other intervention components, while remote completion of self-reported questionnaires was recorded via REDCap. Participants who completed the study protocol including the self-assessment questionnaires received a £25 voucher as compensation.

### Outcomes

Demographic data were collected from the participants’ medical charts. Criteria for feasibility targets were set prior to starting the study and included recruitment rate (≥70%), adherence (≥70% completed three or more modules), completed self-reported baseline and follow-up questionnaires by at least 90% of those who signed informed consent and completers, respectively. We aimed for an attrition rate ≤ 35% (1—% who followed through all the modules). For acceptability, we defined a total score ≥ 30 in Credibility/Expectancy Questionnaire (CEQ)^33^ in ≥70% participants prior to the intervention (T0). Satisfaction was measured with a 5-point Likert scale for overall satisfaction with the treatment at T1 and the Client Satisfaction Questionnaire (CSQ-8),^34^ particularly whether ≥70% would recommend the intervention to others. Unwanted effects of the intervention were evaluated using the 20-item Negative Effects Questionnaire,^35^ aiming for total number of negative effects reported by all the participants/total number of negative effects possible ≤10%. Usability at T1 was evaluated using the sum score and individual item responses of the mHealth app usability questionnaire usefulness subscale.^36^

Several symptom measures were examined at T0 and T1: Metamemory in Adulthood Questionnaire,^37^ subjective Memory Complaint 5-point Likert scale, Brief Illness Perception Questionnaire,^38^ Behavioural Responses to Illness Questionnaire: subscales ‘limiting behaviour’ corresponding to excessive and ‘all-or-nothing behaviour’,^39^ Compensatory Cognitive Strategies Questionnaire,^40^ Patient Health Questionnaire-9 and Generalized Anxiety Disorder-7 scales,^41,42^ EuroQol 5-Dimension-5-Level Health Scale (EQ-5D-5L) five dimensions including mobility, self-care, usual activities, pain/discomfort and anxiety/depression^43^ and work and social adjustment scale^44^ (Supplementary Table 3). Participants also completed a self-rated single-item clinical Global Clinical Impression of Change scale post-intervention.^45^

User experiences were investigated with an open-ended question in the post-intervention questionnaire ‘Let us know what you thought about the programme and the study’ and with semi-structured interviews. An interview guide is available in Supplementary Table 8.

### Sample size

We established a pragmatic sample size target of 30 patients, which is generally recommended for pilot and feasibility studies to provide adequate data and precision of means and variances to answer feasibility questions and inform a fully powered RCT.^46^

### Statistical analysis

Demographics, app engagement and outcomes were summarized using frequencies, percentages, means, medians, standard deviations or IQRs. Mean differences from baseline to post-treatment were analysed with paired *t*-tests or Wilcoxon signed-rank tests based on data distribution. Effect sizes (Hedges g) and confidence intervals (CIs) were reported. Missing follow-up data were not imputed; only available information was analysed.

Attrition was examined by comparing baseline demographics and clinical characteristics across those who did not start, started but did not complete and those who completed the intervention. The association between treatment credibility/expectancy and outcome changes was analysed, dividing participants by the 75th percentile of credibility/expectancy scores and examining with Wilcoxon rank sum tests. Analyses were performed in R (v4.3.1), with a significance level of 0.05.

### Qualitative data

Interviews were carried out via Zoom by V.C. and were recorded and transcribed. The main codes were derived using inductive and reflexive thematic analysis^47,48^ by V.C. using the NVivo V.12 software. V.C. and L.F. identified and discussed the major themes and subthemes and refined these with feedback from the co-authors. A separate framework was built with three categories (positive and negative feedback and suggestions for improvement).

## Results

### Feasibility, acceptability and credibility

Of the 49 participants who were screened for eligibility, 38 met the study’s inclusion criteria and provided informed consent. Thirty-seven participants completed all baseline questionnaires, corresponding to a recruitment rate of 97%. Of these, 30 started the intervention, meeting our recruitment target. Four (13%) participants discontinued the study, so follow-up data were available for 26 (87%) at 6 weeks. Reasons for withdrawing from the study were no time or interest in using the programme (*n* = 2), experiencing functional (dissociative) seizures *de novo* that became the main symptom (*n* = 1) and starting face-to-face psychotherapy (*n* = 1). During the study, three participants experienced technical issues such as forgetting the password and difficulty finding the programme, which were solved by email. Missing data at follow-up were almost entirely due to attrition, rather than difficulties completing the remote online questionnaires. Figure 1 provides a detailed overview of participant flow throughout the study.

Participant baseline characteristics are shown in Table 1. Twenty (54%) participants were female. Mean age at recruitment was 50.4 (*SD* 11), and age at symptom onset was 46.6 (*SD* 11). While all suffered from distressing cognitive symptoms and perceived a decline from their baseline, self-rated memory was very poor or below average in 79% of participants. Only 54% were working full-time, with 22% reporting being unable to work due to cognitive symptoms. Quality of life in the EQ-5D-5L VAS was rated as equal or below 50 by 57%. On the work and social adjustment scale, 89% reported any degree of functional impairment with 22% scoring above 30 (severe impairment). Patients reported that symptoms severely impaired their life (average of 8 out of 10 in the brief illness perception questionnaire, 10 is worse).

**Table 1:** Baseline participant characteristics

Characteristics	Self-help digital therapy (*n* = 37)
Age at recruitment, mean (SD), yrs	50.4 (11)
Age at symptom onset, mean (SD), yrs	46.6 (11)
Symptom duration, mean (SD), yrs	3.8 (3)
Gender	
Female	20 (54)
Male	17 (46)
Self-reported educational level	
High school level or less	19 (51.3)
College degree or equivalent	12 (32.4)
Graduate degree	6 (16.2)
Employment status	
Working full-time	20 (54)
Working part-time	2 (5.4)
Unable to work due to symptoms	8 (21.6)
Unemployed	2 (5.4)
Retired	4 (10.8)
Studying	1 (2.7)
Living situation	
With partner and children	13 (35.1)
With partner	15 (40.5)
With children or other relatives	3 (8.1)
Alone	6 (16.2)
Self-rated memory	
Very poor	14 (38)
Below average	15 (41)
Average	7 (19)
Above average	1 (3)
Excellent	0
Mood disorder	23 (62.2)
ADHD/ASD	6 (16.2)
Functional symptoms and disorders	18 (48.6)
Tremor (*n* = 2)	
Motor and sensory FND (*n* = 3)	
Fatigue (*n* = 12)	
Dissociation/seizures (*n* = 2)	
Dizziness/PPPD (*n* = 4)	
Chronic pain (*n* = 5)	
IBS (*n* = 1)	
Functional jerks (*n* = 1)	
Other comorbidities^a^	30 (81)
Significant life stressors	17 (45.9)
Family history of cognitive symptoms	9 (24.3)
Cognitive screening tests	
Addenbrooke’s cognitive examination (*n* = 12), mean (SD)	85.3 (12)
MoCA (*n* = 4), mean (SD)	26 (1.6)
Missing (*n* = 21)	
Brain MRI scan	
Normal	26 (70.3)
Minor sequelae, unlikely related	1 (2.7)
Missing	10 (27)

ADHD/ASD, attention deficit hyperactivity disorder/autism spectrum disorder.

^a^Migraine (*n* = 8), PTSD (*n* = 7), cancer (*n* = 6), anxiety and panic (*n* = 4), arthritis (*n* = 4), hypertension (*n* = 3), diabetes (*n* = 2), autoimmune diabetes (*n* = 1), hearing loss (*n* = 3), mild traumatic brain injury (*n* = 3), primary biliary cholangitis (*n* = 1), obstructive sleep apnoea (*n* = 2), obstetric antiphospholipid syndrome (*n* = 1), endometriosis (*n* = 1), dilated cardiomyopathy (*n* = 1), asthma (*n* = 2), REM parasomnia (*n* = 1), POTS (*n* = 1), anaemia (*n* = 1), ulcerative colitis (*n* = 2), small fibre neuropathy (*n* = 1).

Of the included participants, the median number of modules completed was 7 (0–7); 23 (77%) completed at least three modules of the programme, meeting the predefined adherence criteria (Table 2); 17 (57%) completed all seven modules; 19 (63%) completed the six core modules; two (6.6%) did not complete any module. The median number of days logged in to the intervention was 8.5 (2–42) (Supplementary Fig. 2). Two (6.6%) participants used the app every day during the study period.

**Table 2:** Feasibility benchmarks

Feasibility outcome	Target set	Observed value
Recruitment of intended participants (*n* = 30)	≥70%	100%
Recruitment rate of those eligible	-	97% (37/38)
Self-completion of online questionnaires		
Baseline—% of completed questionnaires among those who signed informed consent	≥90%	97% (37/38)
Completion of post-intervention online assessments (T1) among those who completed the intervention	≥90%	100% (26/26)
Successful adherence (completing three or more modules and tasks)	≥70%	77% (23/30)
Credibility and expectancy with the intervention (a total score ≥ 30 CEQ, higher scores representing higher credibility/expectancy)	≥70%	68% (25/37)
Client satisfaction questionnaire (would recommend it to others)	≥70%	81% (18/22)
Attrition rate (1—% who followed through all the modules)^a^	≤35%	37% (19/30)
Negative effects: total number of negative effects in the 20-item NEQ reported by all the participants/total number of negative effects possible	≤10%	16% (total number) 5% of treatment-related effects (27 effects reported by seven patients)

CEQ, creditability expectancy questionnaire (T0); NEQ, negative effects questionnaire (T1).

^a^Participants were instructed to complete the six core modules (one per week) with module 7 (on sleep, pain, and fatigue) being optional.

There were no statistically significant differences across any of the baseline characteristics between the participants remaining in the study at the following time points: baseline, programme commencement and intervention completion apart from symptom duration (longer in those who started the programme but did not complete follow-up questionnaires, *P* = 0.04) and medical comorbidities which were more common in those who completed the study protocol (*P* = 0.009) (Supplementary Table 4).

**Figure 2:**
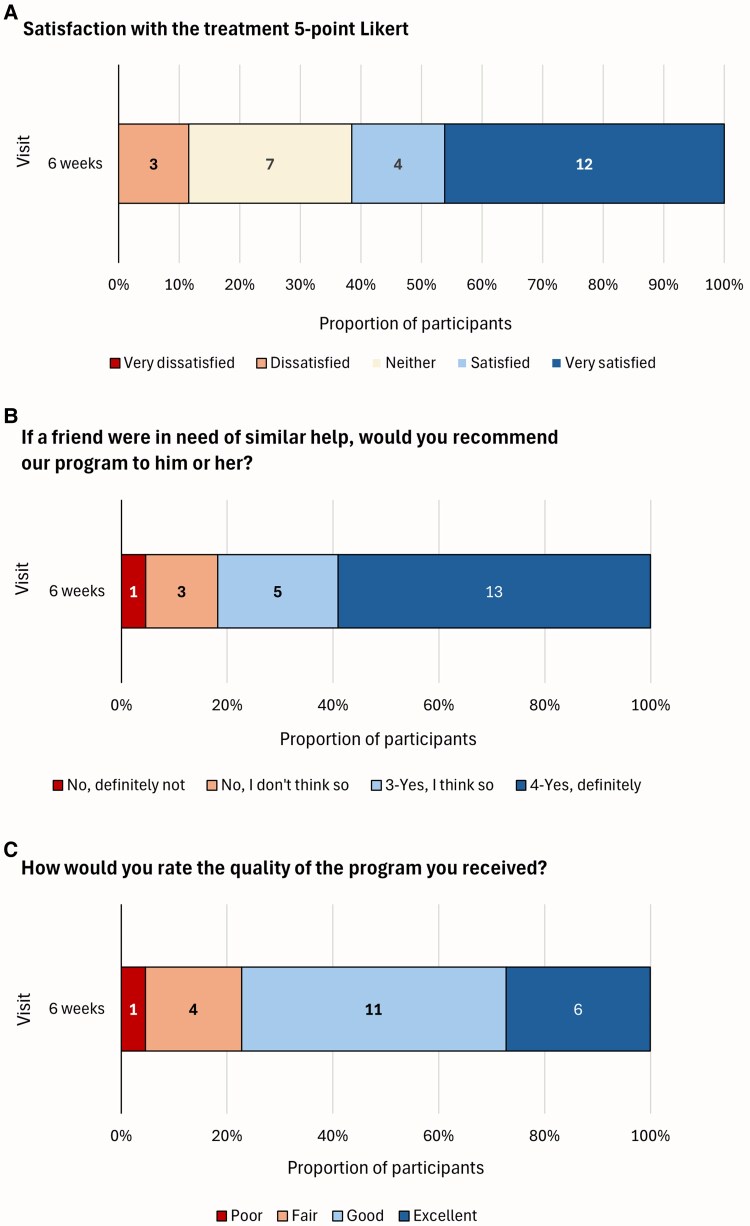
**Participants’ feedback.** Satisfaction with the treatment (**A**) and individual items of the clinical satisfaction questionnaire (**B** and **C**).

Overall satisfaction with the programme was high with 62% (*n* = 16) reporting feeling ‘satisfied’ or ‘very satisfied’ (Fig. 2). Participants generally reported positive experiences (46–73%) across items of the clinical satisfaction questionnaire, with a median score of 27 out of 32, indicating high satisfaction. Among 22 respondents, 82% would recommend the programme to a friend, and 77% rated the programme quality as ‘Good’ or ‘Excellent’. Additionally, 86% would return if they were to seek help again. Immediate feedback of the app modules provided in the app showed high satisfaction, with 88–100% of respondents finding the content helpful (Supplementary Tables 5 and 6).

### Usability and credibility

**Figure 3:**
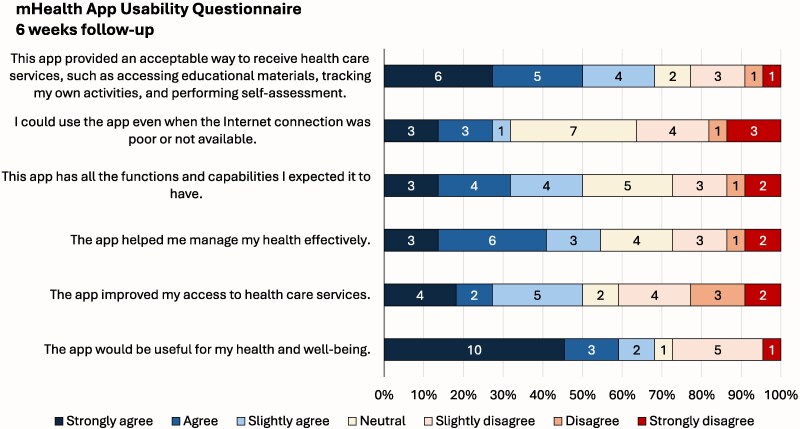
mHealth app usability questionnaire.

At follow-up, we obtained a median score of 19 (6–39, maximum is 42, with higher values representing lower usability) in the mHealth app usability questionnaire. Of the 22 respondents, 16 (73%) found the app useful for their health and well-being and 15 (68%) found it an acceptable way to receive healthcare services (Fig. 3).

Prior to starting the intervention, the mean credibility rating was 17.9 (10–24), and the mean expectancy rating was 13 (6–23) indicating moderate credibility/expectancy (27 representing the maximum score). Expected benefit and perceived credibility prior to starting the intervention did not appear to influence the outcome change (Supplementary Fig. 3).

### Negative effects

Fifteen patients (58%) reported at least one negative effect, mainly attributed to other circumstances external to the treatment (Supplementary Table 7). Seven patients (27%) reported treatment-related negative effects (27 in total, 5% of total number of negative effects possible), comprising ‘I did not have confidence in my treatment’ (*n* = 5), ‘I felt that my expectations were not fulfilled’ (*n* = 5), ‘I felt that the programme did not produce any results’ (*n* = 4), ‘I was under more stress’ (*n* = 3), ‘I did not always understand my treatment’ (*n* = 3), ‘I felt that this treatment was not motivating’ (*n* = 3), ‘I stopped thinking that things could get better’ (*n* = 2), ‘I experienced more anxiety’ (*n* = 1) and ‘I started thinking that my cognitive problems could not get any better’ (*n* = 1).

### Memory diary and symptom check-in entries

Twenty-five (83%) participants used the memory diary and recorded 201 entries (median of entries per patient was 8.1). The median self-rating of memory function across the study was 3 (out of 5). In the symptom check-in, there was a wide variation in individual participant symptom burden with a median number of symptoms of 3 (min–max 1–8) out of 103 entries. The three most frequent symptoms reported were ‘mind going blank’, ‘feeling zoned out or spaced out’ and ‘difficulty sleeping or sleeping excessively’ (Supplementary Fig. 4).

### Preliminary efficacy

At 6 weeks, 16 (62%) reported improvement in their cognitive symptoms (‘minimally improved’, ‘much improved’ and ‘very much improved’). Eight (31%) participants reported ‘no change’. Two (8%) participants reported feeling ‘minimally worse’ or ‘much worse’; both participants scored high for depressive symptoms, with severe and moderate depression, respectively, and experienced PTSD symptoms, with the latter developing dissociative seizures during the study period (Supplementary Fig. 5).

**Figure 4:**
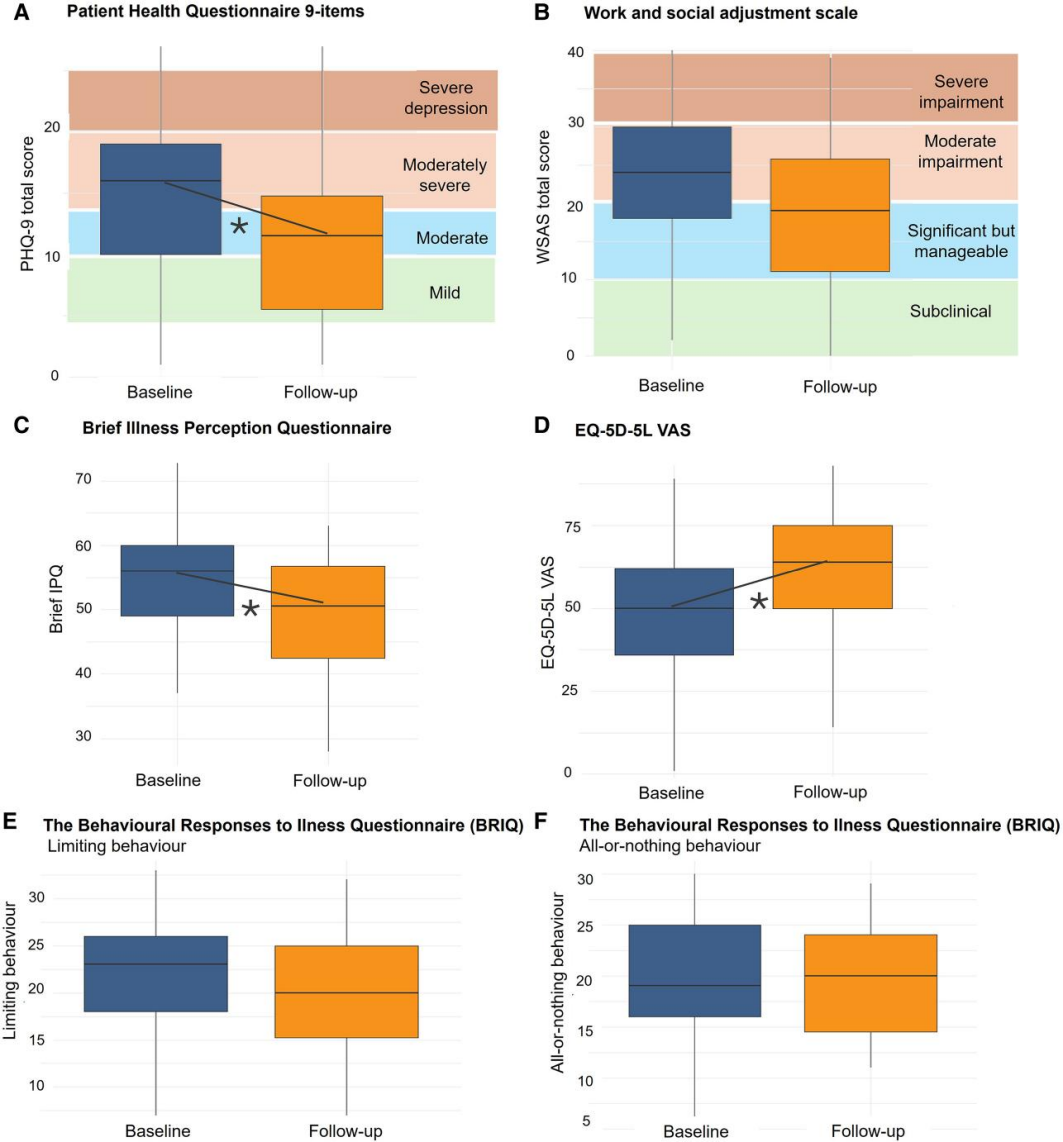
**Change in clinical outcomes between baseline and 6 weeks (paired *t*-tests).** Box plots representing median, lower, and upper interquartile scores. Asterisk (*) indicates statistically significant improvement (*P* < 0.05). (**A**) Depressive symptoms (PHQ-9) (mean change −2.96, Hedges g = 0.40; *P* = 0.008). (**B**) Work and social functioning (mean change −2.92, Hedges g = 0.28; *P* = 0.05). (**C**) Brief illness perception questionnaire (mean change −6.65, Hedges g = 0.68; *P* = 0.001). (**D**) Quality of life measured by EQ-5D-5L VAS (mean change 10.06, Hedges g = 0.48; *P* = 0.03). No significant difference was observed for behavioural responses to illness questionnaire subscales (**E** and **F**) and other symptom measures. A reduction in scores corresponds to a clinical improvement across all scales except EQ-5D-5L VAS, where higher values mean higher quality of life (0–100). EQ-5D-5L VAS: 5-level EuroQoL 5-dimensions visual analogue scale; IPQ: illness perception questionnaire; PHQ-9: Patient Health Questionnaire; WSAS: work and social adjustment scale. *N* = 26.

Descriptive statistics, mean differences in efficacy measures pre-post intervention, effect sizes and their associated values and 95% CIs for clinical outcomes are reported in Table 3. The intervention was associated with improvements in brief illness perception scores (mean change −6.65, Hedges g = 0.68; *P* = 0.001), depressive symptom severity (PHQ-9) (mean change −2.96, Hedges g = 0.40; *P* = 0.008) and quality of life measured by EQ-5D-5L VAS (mean change 10.06, Hedges g = 0.48; *P* = 0.03) (Fig. 4). A detailed analysis of the five individual dimensions of the EQ-5D-5L before starting the intervention and approximately 6 weeks later is provided in Supplementary Fig. 6, showing that more improvements were noted in the usual activities domain of the scale. Improvement in work and social functioning was borderline significant (mean change −2.92, Hedges g = 0.28; *P* = 0.05). While 13 (50%) patients had lower impairment scores in work and social functioning at follow-up, including 4 patients who moved from significant and moderately severe functional impairment to subclinical and 4 patients who were able to return to work, three patients moved to the severe impairment category mainly due to external circumstances (unemployment, son committed suicide, cardiovascular intercurrence) (Supplementary Fig. 5).

**Table 3:** Preliminary effectiveness outcomes (*n* = 26)

	Pre-intervention, mean (SD)	Post-intervention, mean (SD)	Mean change (95% CI)	Hedges g (95% CI)	*P* value
Metamemory in Adulthood Questionnaire (↑)	41 (12)	44 (13)	3.30 (−1.26, 8.87)	0.25 (−0.15, 0.66)	0.15
Compensatory Cognitive Strategies (↓)	62.8 (13)	61.9 (13)	−0.92 (−5.07, 3.22)	−0.07 (−0.47, 0.33)	0.65
Brief Illness Perception Questionnaire (B-IPQ) (↓)	55.3 (9)	48.6 (10)	−6.65 (−10.23, −3.08)	−0.68 (−1.12, −0.23)	**0**.**001**
Behavioural responses to illness questionnaire (↓)					
Limiting behaviours	21.5 (6.8)	19.9 (7.2)	−1.50 (−3.31, 0.31)	−0.21 (−0.61, 0.20)	0.10 0.4
All-or-nothing behaviours	20.3 (5.8)	19.5 (5.2)	−0.77 (−2.73, 1.19)	−0.14 (−0.54, 0.27)	0.033
Patient Health Questionnaire-9 (PHQ-9) (↓)	14.5 (6.9)	11.5 (7.3)	−2.96 (−5.08, −0.84)	−0.40 (−0.82, 0.02)	**0**.**008**
Generalized Anxiety Disorder (GAD-7) (↓)	10.8 (6.3)	9.8 (6.5)	−0.92 (−2.82, 0.97)	−0.14 (−0.54, 0.27)	0.33
Work and social adjustment scale (WSAS) (↓)	21.9 (9.5)	19.0 (10.8)	−2.92 (−5.90, 0.06)	−0.28 (−0.69, 0.13)	0.05
Self-rated health (EQ-5D-5L-VAS) (↑)	49.5 (20.2)	59.5 (20.6)	10.06	0.48	**0**.**03**

↑: higher scores represent improvement; ↓: lower scores represent improvement. Statistically significant differences are highlighted in bold.

There were no significant differences in Metamemory in Adulthood Questionnaire, Compensatory Cognitive Strategies, behavioural responses to illness questionnaire subscales or GAD-7 (anxiety).

### Qualitative findings

Six participants (four women and two men, mean age 53.6 years) participated in a semi-structured interview. Interviews lasted 60 min. The thematic analysis of patient interviews and the post-intervention open-ended question yielded three themes [(i) changing symptom perception, (ii) empowerment and (iii) practical integration of strategies learned] and eight subthemes (Table 4 for themes and quotes and Supplementary Table 8 for a detailed report). Qualitative feedback grouped into positive and negative usability aspects, including suggestions for improvement and future functionality are also summarized in Supplementary Table 9.

**Table 4:** Thematic analysis of questionnaires and semi-structured interviews

Theme	Subtheme	Sample quotes
Changing symptom perception	Thought reframing	*‘It helped me realise that just because I forget things some days it doesn’t mean I will always forget.’* *‘I was always imaging the worst possible scenario. Now I try to think about what would the “ally voice” say and look for alternative explanations.’* ‘*What a weight is lifted by the reassurance that it’s quite normal what I forget.’* ‘*I can now recognize it may not be dementia because if it's dementia by now, I could not be functioning well.’* ‘*It helped me understand the memory process better and that forgetting can also be helpful.’* ‘*This has helped with my perception of what may be going on inside my brain, and I worry much less since.’*
	Regaining confidence	*‘I felt confident enough to try and for the first time in 2–3 years was able to meet with friends and clients in the evening after work.’* ‘*I have adopted a more positive posture and think that I capresenn do certain things, while before I would think that I couldn’t.’*
Empowerment	Being in control	*‘I feel what’s been hugely helpful is accepting that there are things under my control and others I do not control.’* ‘*I used to get stuck more in my conversations, and as soon as I would forget a word, I would be panicking. But now I take a deep breath and I say, hey, it's not big deal.’* ‘*This has reassured me that I can help myself to improve things.’*
	Identity	*‘I am starting to feel like myself again, I can even experience a genuine laugh again.’*
	Symptom validation	*‘I cried when I was listening to the stories of people who have the same experience in dealing with this difficult problem. For the first time I'm actually feeling validated.’* ‘*I could not actually get better because of this, this problem of not being recognized.’*
Practical integration	Convenience	*‘It is great to have all the answers and support in one source.’* ‘*It allows me to work on my perspective and mindset at a time that suits me. I can reinforce the messages as often as I need to.’* ‘*Part of the problem is not knowing where to turn or it being too expensive. So, continuing the treatment at home is great.’*
	Translating learning into practice	*‘I started to pace my activities to try and avoid reaching limit points.’* ‘*This app helped me being more in the present moment and focusing more in the tasks I’m doing.’* ‘*I did the homework task and (…) there was a realisation of ‘I can't improve if don’t communicate with others.’* ‘*I have managed to begin reading books for a short time (…) also joined a volunteer group.’* ‘*I am putting a big effort to rest physically and mentally, avoiding ‘pushing through’.’* ‘*I have started to incorporate ten minutes of yoga into my evenings and getting out walking more. I am benefiting from exploring new hobbies and noticing a difference with my memory.’*
	Barriers	*‘I wrote down the strategies but I forgot where I put them, didn't remember why I wrote them and felt that I needed to revisit the app again to reread.’* ‘*The service was not personalised and didn't help me with my personal circumstances.’* ‘*I had all symptoms together but no supportive environment, so I am living with more stress and anxiety.’* ‘*I had multiple notifications from the app but my problem was always remembering to use it.’* ‘*I liked the programme and will continue to use it, but I memorise the programme better in a longer period than six weeks. I think the programme is perfect for longer use.’*

Usability findings and suggestions for future improvements are reported in Supplementary Table 9.

## Discussion

Mementum, a novel 6-week self-help digital programme for FCD, met most of the feasibility targets, including satisfaction with care, acceptability and adherence. The 77% adherence rate was probably superior to those reported in research trials testing self-help internet-based interventions (adherence rates ranging from 2 to 92%)^49^ and comparable to face-to-face interventions. Most participants continued using the app beyond the initial 2 weeks, a common dropout point in self-help apps.^50^ Although the increased support and motivation inherent to a research study may partially account for this, this is significantly better than a median retention rate 3% at 30 days reported by a review of 93 mental health apps.^49,51^ Additionally, 87% completed the 6-week assessment, compared to just 3.5% in a similar self-guided CBT-based app (Happify).^49^ Attrition was mainly due to external factors that overlap with those observed for face-to-face interventions (e.g. change in symptom/personal circumstances and content not as expected), moving to other interventions, or dissatisfaction with the programme. Those with longer symptom duration were more likely to interrupt the study, but we also noticed functional improvements in work and social domains in patients with lengthier symptom history. The absence of differences in most clinical and sociodemographic characteristics in adherence to the treatment protocol suggests that this self-guided intervention can probably be used by most individuals with FCD, perhaps except for those with reduced social and therapeutic support, which generally conveys a poorer prognosis in functional disorders.^52^

The human-centred design combining ‘persuasive system design’ (e.g. notifications) and ‘behavioural economics’ (e.g. gamification and completion badges) techniques may have facilitated engagement and enhanced the app clinical effects.^53,54^ Based on patients’ feedback, symptoms varied widely and the possibility of tailoring the modules to their main symptoms made the content relevant to a range of users, which is crucial to pursue in real-world implementation. However, some did not find the app helpful, and their participation in the study temporarily resulted in heightened anxiety, reduced work and social functioning or symptom worsening. While this response is somewhat expected with interventions aimed at behavioural modification, particularly among those who adopted avoidance of cognitive activities and/or social function as their coping strategy, high baseline levels of anxiety and depressive symptoms, other comorbidities, busy life schedules and stressful life events are likely to have contributed. Plus, any form of online and offline education requiring patients to track, read about or engage with their symptoms may amplify symptomatology. Similarly, additional human support (calls and emails) was experienced as overwhelming to some patients who deemed the automatic support sufficient with the possibility of contacting for technical support, if needed. This suggests that the use of self-guided interventions as a first-step treatment approach is suitable, facilitating shared decision-making and allowing patients to select the alternative they may find more effective and acceptable for their circumstances. It has further practical implications as factors other than human guidance, including framing the intervention as self-help within the context of a clinical encounter, a deadline effect^55^ and a user-friendly interface^56^ optimized with users’ feedback seem to be key factors contributing to treatment success, possibly without compromising therapeutic alliance.^57^ Although for some, the lack of guidance and personalization was certainly a limitation, the remote intervention was deemed as convenient (e.g. by minimizing work absences and travelling costs and offering the possibility of revisiting content as needed), suggesting that a full-scale RCT using the same study methods, including automated support options to enhance motivation and adherence,^58^ is worth pursuing.

More than half of the completers reported symptom improvement. The intervention was associated with significant small-to-moderate pre-post treatment effects in symptom burden, namely, improvements in quality of life, depressive symptoms and illness representations. It is possible that ‘treatment effects’ merely reflected natural symptom recovery or regression to the mean. Similarly, an influence of research team care, including a clear explanation and support, cannot be ruled out, even though it was limited to a brief contact of approximately 1 h before completing the intervention. These and the so-called digital placebo effect^54^ are also possible. Yet, we did not find associations between the expectancy for the treatment prior to starting the intervention and the observed outcome changes. Plus, this is counteracted by the fact that FCD symptoms tend to remain chronic if left untreated and the mean duration of symptoms at the time of enrolment was 3.8 years. Our data suggest that some of these apparent treatment effects might have been moderated by an improvement in depressive symptoms.

Despite the high satisfaction reported, there was no significant improvement in memory symptoms as measured by the Metamemory in Adulthood Questionnaire and many still rated their memory as ‘poor’ or ‘below average’ after the intervention. While either the intervention or its implementation may have failed to address memory symptoms effectively, it is also likely that the outcome measures might have not been suitable to capture the metacognitive biases often present in FCD^59^ and the multidimensional nature of the symptoms, echoing the challenges identified in previous trials in functional disorders.^19,60,61^ It is also possible that a longer follow-up period is necessary to detect changes in memory symptoms beyond the 6 weeks. Reviews of other digital interventions suggest that users’ perceptions of benefit do not always align with quantitative benefits. In fact, recent research corroborates the impression that patients’ perception of improvement often goes beyond symptom-based scales and reflects other sentiments such as feeling more equipped to deal with symptoms and less threatened by them.^62^ For instance, increased confidence in the diagnosis, reduced symptom-related anxiety and a willingness to engage in cognitive activities may potentially pave the way for further recovery. The intervention was also beneficial to validate symptom experiences, addressing a key user need for accessible and credible information.^63^ While no other self-help digital interventions specific for FCD are available for direct comparison, similar self-guided interventions have shown small-to-moderate improvements in quality of life and symptom reduction in conditions like chronic pain, chronic fatigue, anxiety and depression.^53,64-67^ Similar trials of low-intensity structured CBT-based strategies often face similar challenges in balancing standardization with personalization, leading to unwanted heightened anxiety and isolation feelings in some patients.^68^

Qualitative feedback further suggested that the different treatment approaches featured in our intervention potentially benefited different patient profiles in varying degrees and may have reinforced strategies learned. Specifically, initial education around attention and the mechanisms underlying FCD, followed by cognitive restructuring and cognitive rehabilitation strategies, may have provided a bridge to support engagement in previously avoided cognitive activities while also reinforcing exposure to memory successes that allow FCD patients to progress in their recovery journey. This is supported by a recent small trial in FCD post-concussion comparing CBT and cognitive rehabilitation strategies which found similar benefits in both arms.^11^ This intervention will be further improved based on users’ feedback and other scientific findings that may become available in the future. Taken together, data from this study add new insights over the needs and symptoms of FCD patients and the risks associated with interventions for FCD, while also highlighting the need for a thorough eligibility assessment of this population covering other neurological symptoms, anxiety and depression and social circumstances which can be expected to fluctuate over time and affect intervention adherence. This knowledge will help select which patients are more likely to benefit from this intervention, while ensuring that others are offered adequate support for their symptoms. Future iterations of this intervention should also focus on improving treatment credibility and minimizing potential negative effects.

### Limitations

This study has several limitations. First, the study is a non-randomized pilot evaluation of a novel digital intervention, it did not evaluate the willingness to randomization and its findings need to be interpreted with caution, given the small sample size and the absence of a control group. Its main goal was to provide feasibility data and inform a future RCT with a head-to-head design. The potential effects of medical attention and pharmacotherapy were not controlled for as participants continued to receive usual care. The lack of standardized cognitive measures may be considered a limitation by some, but this was intentional as FCD patients may demonstrate impairment in testing,^69^ and test performance can be fluctuating and often does not translate well to daily functioning. The follow-up period was relatively short and long-term effects were not assessed, so delayed benefits might have been missed and durability of the effects cannot be determined. Given that several patients chose not to start the intervention after consenting and filling in the baseline questionnaires, selection bias may have occurred at this level. We also cannot exclude that some FCD patients had in fact another diagnosis or may develop neurodegeneration in the future, particularly considering that not all patients underwent formal cognitive assessment and neuroimaging at baseline.

## Conclusion

The present study shows that this first self-help digital intervention for FCD is feasible, acceptable, safe and well received by the majority of patients, as a first-step approach part of a stepped care model for FCD. A broad range of feasibility outcomes, engagement metrics and qualitative feedback were analysed in this mixed-methods study, to account for the heterogeneity of the target group and to ensure that all procedures and the intervention are optimized according to participants’ experience prior to a future RCT. Despite the barriers and limited efficacy in certain groups, app-based treatment may offer accessibility for under-served patients for whom a self-help resource might be the only option to receive health support, with the potential of leading to an improvement in clinical outcomes in a flexible and cost-saving manner. Given the prevalence of FCD, this may still benefit patients in the future who have no other therapeutic support. Although a full-scale efficacy trial is needed to replicate and extend on these preliminary findings, we anticipate that this novel intervention may be potentially complementary to other guided interventions currently under development and more intensive personalized multidisciplinary interventions involving neuropsychiatry, speech and language therapy and/or occupational therapy.^70^

## Supplementary Material

fcaf248_Supplementary_Data

## Data Availability

Data are available from the authors upon reasonable request.
