# Supplementary material for: Feasibility trial of a self-help digital intervention for functional cognitive disorder
Source: Brain Commun. 2025 Jun 19;7(4):fcaf248. doi: 10.1093/braincomms/fcaf248 (PMC12241858; doi:10.1093/braincomms/fcaf248)
Supplement: fcaf248_Supplementary_Data [file fcaf248_supplementary_data.docx]

**Supplementary material**

**Supplemental Table 1.** Detailed overview of the contents of Mementum modules and tasks(1).

| Module | Content | Tasks |
| --- | --- | --- |
| 1 | Education on cognitive symptoms, memory processes and importance of attention to encoding and retrieval of memories;  Recognition of attention as a limite resource and as a a filter of other stimuli (e.g. show that we can’t assess resources under pressure).  Education about normal memory lapses, portraying them as part of normal human experience. | Noticing other’s memory failures.  Collect others’ opinions about one’s memory failures. |
| 2 | Education about functional cognitive disorder regarding its frequency, rationale for diagnosis, common symptoms and mechanisms, including vicious loop driven by self-monitoring, negative illness perceptions and maladaptive behaviours.  Contextualisation of functional cognitive disorder with potential bio-psycho-social vulnerabilities, triggers and perpetuating factors.  Addressing performance anxiety and fear of failure. | Recognition of own’s positive performance/achievements over the last week to promote recognition of cognitive successes. |
| 3 | Exploration of the relationship between thoughts and symptoms including the importance of refocusing attention on tasks in hand instead of focusing on symptoms and thoughts.  Presentation of alternative helpful thoughts using patient narratives - practicing an “ally” voice that supports positive thinking.  Alternatively, techniques to create a distance to one’s thoughts. | Recognition of potential triggers of cognitive symptoms and altered thoughts with exploration of alternative thinking patterns and helpful responses. |
| 4 | Education on short- and long-term consequences of engaging in defensive behaviours such as avoidance (e.g. giving up hobbies and tasks) and safety strategies (e.g., repetitive checking) that might interfere rather than promote functioning.  Inspire participants to overcome the fear of failing by “trying their brain out”: getting away from avoidant loops, including cogniphobia. | Stimulate progressive challenge (starting by doing things less perfectly) and behavioural analysis. |
| 5 | Specific techniques to facilitate symptom management, increase self-confidence in memory and rehabilitative/compensatory strategies.  Specific techniques focusing on distraction, multi-tasking and intrusive thoughts. Techniques to promote active noticing and implemented intentions to help with prospective memory tasks (e.g. intentional encoding, repetition, taking breaks, management of distractions, FOUR technique to facilitate encoding).  Introduction of habits and routines, and pros and cons of calendar use.  Occupational therapy and speech and language techniques for specific symptoms e.g. word-finding difficulties. | Practicing and experiment learned techniques in daily life. |
| 6 | Introduction to the relationship between major stressors and life events, including the cognitive effects of stress (individual variability, “low stress threshold”, and self-perpetuating cycle of stress).  Helping the person understand physical symptoms and cognitive symptoms due to anxiety and depression (concept of brain-mind-body disconnection).  Cover emotional reactions to the symptoms (neurocognitive-specific health anxiety/hyperarousal, anger, frustration, blame, self-criticism) to increase emotion recognition. | Identification of own’s values and main goals: re-direct energy to activities that bring higher satisfaction.  Offering opportunities to integrate valued activities into daily life to achieve balance and counteract “boom and bust” patterns; |
| 7 | Sleep hygiene education, stimulus control and techniques on sleep restriction.  General advice on exercise, symptom control (e.g., pain and fatigue), healthy diet and medication management. | - |

**Supplemental Table 2.** Intervention interactive features(1).

| **Registration process** | Users are provided with a personal voucher code and register with an email address and password.  Acceptance of program’s terms and conditions for use.  Access to the program is automatically provided for six weeks. |
| --- | --- |
| **Onboarding** | Introduction to the programme and app functions, reinforce the need for commitment for a successful outcome out of the programme, show clear expectations.  Provide value and make users excited from the start.  Initial memory diary and symptom checklist registration.  Ensure credibility of the intervention (evidence-based and developed by healthcare professionals) |
| **Search bar** | Topical selection based on users’ search. |
| **Favourites** | Option to save content that they have already completed and found relevant. |
| **Tailoring** | Content can be suggested based on their search and main symptoms reported in the symptom check-in. |
| **Feedback question** | After completing each module, users answer a feedback question regarding how helpful it was. |
| **‘My results’** | In the user profile, this area provides a summary of the progress in the programme and memory diary/symptom check-in entries over the 6 weeks. |
| **Gameification** | 1. ‘Up next’: dashboard feature where users can see a list of tasks to complete next in the programme according to a pre-defined setting.  2. Progress bar during activities.  3. Virtual ‘badges’ as a reward for module and programme completion.  4. Day counter: dashboard feature allowing users to track their days in the programme. |
| **Mindfulness video exercises** | Examples of exercises available include:   - Attention training exercises - Deep-breathing - Progressive muscle relaxation - Mindful meditation and walking - Grounding and centering - Practice of exercises on demand |
| **Patient stories menus** | Six patient stories (text and audio recordings) can be accessed directly in one place. |
| **Memory diary**  **Symptom check-in** | Memory diary intended to keep a log of symptoms and increase memory self-efficacy and promote a sense of progress.  Symptom check-in provides an opportunity to tailor content to users’ symptoms.  Adjunctive push and in-app notifications to prompt completion of these questionnaires sent once a week. |
| **Homework tasks** | One to complete each week related to a specific module.  Users can experience a sense of accomplishment by ticking off tasks. |
| **FAQs** | A link to the study website page on “frequently asked questions” (FAQs) (e.g. why am I not getting better?’ or ‘How to communicate my symptoms to others?’). |
| **External link resources** | A link to the study website page on external link resources, which contain further information on managing other symptoms such as pain, fatigue and insomnia. |
| **Contact section** | Option for users to write a message to the research team in the study website. |
| **“Persuasive design” to promote contact with the intervention** | Push notifications once a week if users did not login to the app (link to modules and memory diary)  Automatic emails after completing each module to promote continuous engagement and links to other content. |
| **Technical support** | Offered by the research team via email or telephone calls. |

**Supplemental Table 3.** Study Questionnaires and ranges.

| **Questionnaire** | **Scoring** |
| --- | --- |
| **Metamemory in Adulthood Questionnaire measures metamemory or subjective memory and includes subscales capacity (perceived everyday memory performance), change (perceived change in memory) and memory-related anxiety(2)** | Higher scores reflect greater knowledge and better memory capacity. |
| **Brief Illness Perception Questionnaire assesses the cognitive and emotional representations of illness (treatment target with CBT)(3)** | Maximum score of 80, with score <42 representing low threat, 42-49 intermediate threat levels, and $\geq50$ representing high threatening perceptions. |
| **Compensatory Cognitive Strategies Questionnaire (CCSQ)(4)** | Higher scores represent more everyday cognitive strategies used (e.g. diary use). |
| **Patient Depression Questionnaire (PHQ-9)(5)** | Total score ranges from 0 to 27 with scores of 5–9 classified as mild depression; 10–14 as moderate depression; 15–19 as moderately severe depression; and ≥ 20 as severe depression. |
| **Generalised anxiety disorder scale (GAD-7)(6)** | Score ranges from 0-21: score of 0–4 corresponding to no anxiety, 5–9 (mild), 10–14 (moderate), and 15–21 (severe anxiety). |
| **Behavioural Responses to Illness Questionnaire subscales "limiting behaviour" corresponding to excessive rest items and "all-or-nothing behaviours"(7)** | Higher scores representing more behavioural responses to illness. |
| **Work and Social Adjustment Scale (WSAS)(8)** | Score ranges from 0-40, with higher scores indicating more severe functional impairment: 0-10: Subclinical impairment; 11-20: Significant functional impairment, but manageable; 21-30: Moderately severe functional impairment; 31-40: Severe functional impairment. |
| **EuroQol 5-Dimension-5-Level Health Scale (EQ-5D-5L) five dimensions including mobility, self-care, usual activities, pain/discomfort and anxiety/depression(9)** | Five levels range from no problems to extreme problems. |
| **EQ-5D-5L visual analogue scale(9)** | 0 to 100 with higher scores indicating better overall health (quality of life). |
| **Subjective Memory Complaint 5-point Likert scale** | 5-points ranging from 1 (very poor) to 5 (excellent) |
| **Clinical Global Impression-Improvement Scale, single item, participant rated(10)** | 7 items ranging from 1 (Very much improved) to 7 (Very much worse) |
| **Client Satisfaction Questionnaire(11)** | 0 to 32, with higher scores indicating more satisfaction |
| **Satisfaction with the treatment 5-point Likert scale** | 5-points ranging from 1 (very satisfied) to 5 (very dissatisfied) |
| **Credibility expectancy questionnaire(12)** | Two subscales ranging from 3 to 27 (items presented in % were transformed to a scale from 1-9 to allow for composite scores to be obtained) |
| **mHealth usability questionnaire usefulness subscale(13)** | 7 points scale, higher scores indicate poorer usability |
| **Negative effects questionnaire(14)** | 20-item questionnaire, with items divided into treatment-related versus other circumstances-related effects |

| **Supplemental Table 4.** Comparison between participants who consented but did not start the intervention, those who started but did not complete post-intervention assessment, and those who completed the protocol. | | | | |
| --- | --- | --- | --- | --- |
|  | **Consented but did not start (n=7)** | **Missing post-interventions assessment (n=4)** | **Completed post-interventions assessment (n=26)** | **P value** |
| Age at recruitment, mean, y | 47.6 | 45.8 | 51.9 | 0.32 |
| Female sex, N(%) | 3 (43) | 3 (75) | 14 (54) | 0.68 |
| Symptom duration, mean, y | 3.4 | 8.3 | 3.6 | **0.04** |
| Self-rated memory (5-point Likert scale) | 1.7 | 1.5 | 2 | 0.55 |
| Medical comorbidities, N(%) | 3 (43) | 3 (75) | 24 (92) | **0.009** |
| Other FNDs, N(%) | 2 (29) | 4 (100) | 12 (46) | 0.06 |
| Mood disorder, N(%) | 4 (57) | 3 (75) | 16 (62) | 1 |
| Significant life stressors, N(%) | 3 (43) | 1 (25) | 13 (50) | 0.76 |
| Family history of cognitive symptoms, N(%) | 1 (14) | 1 (25) | 7 (27) | 0.85 |
| Living alone, N(%) | 3 (43) | 1 (25) | 2 (8) | 0.06 |
| Employment status (working), N(%) | 4 (57) | 2 (50) | 16 (62) | 0.97 |
| Metamemory in Adulthood Questionnaire | 34.7 | 37 | 41 | 0.39 |
| Compensatory Cognitive Strategies | 60.7 | 61.2 | 62.8 | 0.94 |
| Brief Illness Perception Questionnaire (B-IPQ) | 52.6 | 51.8 | 55.3 | 0.73 |
| Behavioral responses to illness questionnaire  Limiting behaviors  All-or-nothing behaviors | 24  19.6 | 22.5  18 | 21.5  20.3 | 0.66  0.62 |
| Patient Health Questionnaire-9 (PHQ-9) | 13 | 18.5 | 14.5 | 0.27 |
| Generalized anxiety disorder (GAD-7) | 11.3 | 9.75 | 10.8 | 0.94 |
| Work and social adjustment scale (WSAS) | 26.6 | 30.8 | 21.9 | 0.12 |
| Self-rated health (EQ-5D-5L-VAS) | 52.9 |  | 49.5 | 0.91 |
| Credibility (3-27) | 16.9 | 18.5 | 18.1 | 0.74 |
| Expectancy (3-27) | 12.6 | 13.5 | 13.1 | 0.94 |

Baseline: baseline completer. Commencer: programme commencer. Completer: programme completer.

**Supplemental Table 5.** Client satisfaction questionnaire.

| **Client Satisfaction Questionnaire (CSQ) (item range 1-4)** | **Participants answering positively (3-4), n(%)** |
| --- | --- |
| **How would you rate the quality of the program you received?** | 17/22 (77%) |
| **Did you get the kind of help you wanted?** | 18/22 (82%) |
| **To what extent has our programme met your needs?** | 12/22 (55%) |
| **If a friend were in need of similar help, would you recommend our program to him or her?** | 18/22 (82%) |
| **How satisfied are you with the amount of help you received?** | 18/22 (82%) |
| **Have the information you received helped you to deal more effectively with your cognitive problems?** | 17/22 (77%) |
| **In an overall, general sense, how satisfied are you with the service you received?** | 18/22 (82%) |
| **If you were to seek help again, would you come back to our programme/App?** | 19/22 (86%) |

**Supplemental Table 6.** Immediate feedback provided in the app regarding individual modules.

| **Modules** | **N** | **Proportion answering ‘Yes’ to “Did you find this helpful?”** |
| --- | --- | --- |
| **Module 1** | 28 | 100% |
| **Module 2** | 25 | 100% |
| **Module 3** | 23 | 91% |
| **Module 4** | 19 | 95% |
| **Module 5** | 20 | 95% |
| **Module 6** | 17 | 88% |
| **Module 7** | 17 | 94% |

**Supplemental Table 7.** Negative effects questionnaire.

| **Negative Effects Questionnaire (NEQ) items** | **N (%)** | **Treatment related** | **Other circumstances** |
| --- | --- | --- | --- |
| **I had more problems with my sleep.** | 7 (23) | 0 | 7 |
| **I felt like I was under more stress.** | 8 (27) | 3 | 5 |
| **I experienced more anxiety.** | 5 (17) | 1 | 4 |
| **I felt more worried.** | 4 (13) | 0 | 4 |
| **I experienced more hopeless.** | 2 (7) | 0 | 2 |
| **I experienced more unpleasant feelings.** | 4 (13) | 0 | 4 |
| **I felt that the issue I was looking for help with got worse.** | 1 (3) | 0 | 1 |
| **Unpleasant memories resurfaced.** | 4 (13) | 0 | 4 |
| **I became afraid that other people would find out about me using this app.** | 1 (3) | 0 | 1 |
| **I got thoughts that it would be better if I did not exist anymore.** | 1 (3) | 0 | 1 |
| **I started feeling ashamed in front of other people.** | 2 (7) | 0 | 2 |
| **I stopped thinking that things could get better.** | 4 (13) | 2 | 2 |
| **I started thinking that my cognitive problems could not get any better.** | 8 (27) | 1 | 7 |
| **I did not always understand my treatment.** | 5 (17) | 3 | 2 |
| **I think I developed a dependency on the help I got.** | 2 (7) | 0 | 2 |
| **I did not always understand my doctor.** | 3 (10) | 0 | 3 |
| **I did not have confidence in my treatment.** | 7 (23) | 5 | 2 |
| **I felt that this programme and study did not produce any results.** | 5 (17) | 4 | 1 |
| **I felt that my expectations were not fulfilled.** | 5 (17) | 5 | 0 |
| **I felt that this treatment was not motivating.** | 4 (13) | 3 | 1 |

**Supplemental Table 8.** Interview outline.

|  | **Questions** |
| --- | --- |
| **General feedback on the intervention and study** | Overall, how did you feel about the intervention and the study?  Which part did you like most? Why?  Were there any parts that didn’t like?  What would you change?  Is there any other information you would like to have seen in the App? Any other suggestions? |
| **Format and language** | Were there any words you didn’t understand?  What did you think about the format of the content? (videos, text, visuals, audio) |
| **Navigation** | How was the experience of navigating in the app? Can you name positive and negative aspects?  Would you have preferred all the modules open to enable you to search topics and tailor to your symptoms or closed and opening as you go (pre-defined pathway)? |
| **Guidance** | Was self-help acceptable for you? If not, why not?  How did you find the emails, push notifications and phone calls?  Did you prefer telephone/email or it doesn’t matter? |
| **Duration** | What did you think about the duration of the programme?  Did you feel that 6 weeks was adequate, too short or too long? |
| **Questionnaires** | Was it easy to complete the questionnaires? |
| **Engagement** | In your case, how did you keep motivated to engage with the app and study? |

Six participants (four women and two men, mean age 53.6 years) participated in a semi-structured interview. Interviews lasted 60 minutes. Moreover, the 26 participants who completed the post-intervention questionnaires provided a brief written response regarding their overall feedback about the programme and study. Three themes and eight subthemes were identified.

The first theme “*changing symptom perception*” related to the overall treatment model and goals of the intervention. Participants experienced “*thought reframing*” representing positive effects of the cognitive restructuring content and a better knowledge about memory processes, experiencing memory lapses as less worrying and, particularly, being less concerned that any memory slips were a sign of underlying dementia. They also noticed a shift in “*confidence in memory*” which allowed them to start reducing avoidance strategies.

The second theme related to “*empowerment*”, particularly “*being in control*” of the symptoms while simultaneously acknowledging that there are things that cannot be modified. One participant mentioned a reduction in hyperarousal which helped him to take action to overcome word-finding difficulties. Two other subthemes identified were “*identity*” and “*symptom validation*”, both reflecting the impact of the symptoms in the construct of self- and the importance of learning from others’ experiences and feeling “heard and understood”.

The third and last theme relates to “*practical integration*” of the strategies learned. The participants mentioned satisfaction with the convenient character of the intervention, namely the possibility of revisiting content and using the app at a time that suits them. They also mentioned several examples of strategy use in everyday life, including more traditional cognitive rehabilitation strategies, such as active listening, staying calm and taking breaks, signing up for valued activities like volunteering and new hobbies, and incorporating mindfulness moments in their day. Although one participant actively practiced the thought reframing suggestions, most of the feedback was related to practical exercises and behavioural experiments rather than avoidance. This relates to the subtheme “*regaining confidence in memory*”. Several participants reinforced the practice of mindfulness and attention-training exercises by downloading other dedicated apps to these topics.

There were also “*barriers*” that show that factors in the environment and private life may affect experiences, treatment effects and feasibility in both positive and negative ways. Some patients reported difficulties in finding the time to complete the modules in their busy schedule, and an increased anxiety and stress as a result, especially when prompted by the intervention. A few participants noted that they had difficulties remembering to use the programme, or recalling the content of the programme, hampering the ability to apply the strategies or knowledge in their daily lives. The lack of content especially relevant for their individual circumstances and the lack of social support were also viewed as potential barriers to engage in self-help.

The most positive aspects noted were the overall appearance of the app and the possibility of having different resources from information to videos and exercises in one source. Twelve participants found the app informative, helpful, and that the content resonated with their symptoms. The memory diary was appreciated by many, and an expansion of this feature was suggested for visual tracking of progress. Yet, some app users criticized the lack of added value regarding the app's level of detail compared to other sources “*too simplistic*” and “*Much of this can also be found on Google*”. Also on the technical implementation side, many participants welcomed the possibility of browsing freely, saving content in favourites and tailoring the content to their symptoms. Others reported difficulties with navigation or found it less intuitive, especially with back and forward navigation, and would prefer a standalone app for the ease of the download process. As worst usability-related aspects, three participants mentioned that they found the app notifications and reminder emails distracting and a source of additional stress in busy times, which they found counterproductive. Three participants said that they would like to be able to select more than one symptom at once in the symptom check-in. Two participants reported that it wasn’t clear to them what they were expected to do, and two found that the programme content could be potentially more beneficial with long term use. Two patients with prior found the content repetitive and simplistic given their prior knowledge of FCD; these two participants said they would have preferred a face-to-face intervention. One participant with listening difficulties mentioned that all videos should have subtitles, and not just some. Earlier in the study, feedback from participants suggested that a weekly email and a bi-weekly phone call from the research team, in addition to the automatic support from motivational emails and push notifications was overwhelming the participants, and that the participants were managing to complete the programme at their own pace, tailoring their preferred activities to their symptoms. Hence, the phone calls were reduced to twice during the study, and emails were sent only every other week, with a possibility of additional contacts for technical support if needed.

**Supplemental Table 9.** Usability feedback, and suggestions for future improvements.

|  |  | Summary of findings |
| --- | --- | --- |
| Usability | **Positive aspects** | - Participants welcomed the content being unlocked and having the possibility of browsing freely. - Flexibility of tailoring the content to the symptoms. - Overall appearance and calming colour scheme. - Videos with subtitles were welcomed. - Easy access to the team via e-mail was valued. - Acronyms for key messages and visuals facilitated content retention. |
|  | **Negative aspects (needing improvement)** | - Some participants felt that module follow-up emails and reminders were distracting or caused additional pressure/anxiety to complete the programme, and so tended to ignore them. - Prompts would also cause extra stress when then reminded to complete tasks. - Given that multiple symptoms are often present and interconnected, participants would have preferred an option to select multiple symptoms in the symptom check-in. - Navigation should allow to move back and forward in every content type. - The ability to save progress of unfinished modules was requested. - The use of AI-voice was deemed as inadequate by two patients. - Patients would prefer a standalone app to improve accessibility. - Participants with prior knowledge of FCD found some sections a bit simplistic and repetitive. - Glitches retract from engagement. - Font size should be bigger in images and videos. |
|  | **Suggested programme improvements (future directions)** | - Participants suggested further ways to enable track symptom progress, perhaps with visual representation of memory diary results. - More patient recovery stories were welcomed and requested. - Participants would like to hear about research updates and other treatments options via the support website. - A forum to connect with other patients was suggested. - Various other relaxation strategies were suggested. |

**Supplemental Figure 1.** Overview of the content of Mementum intervention. FAQs: frequent asked questions(1).


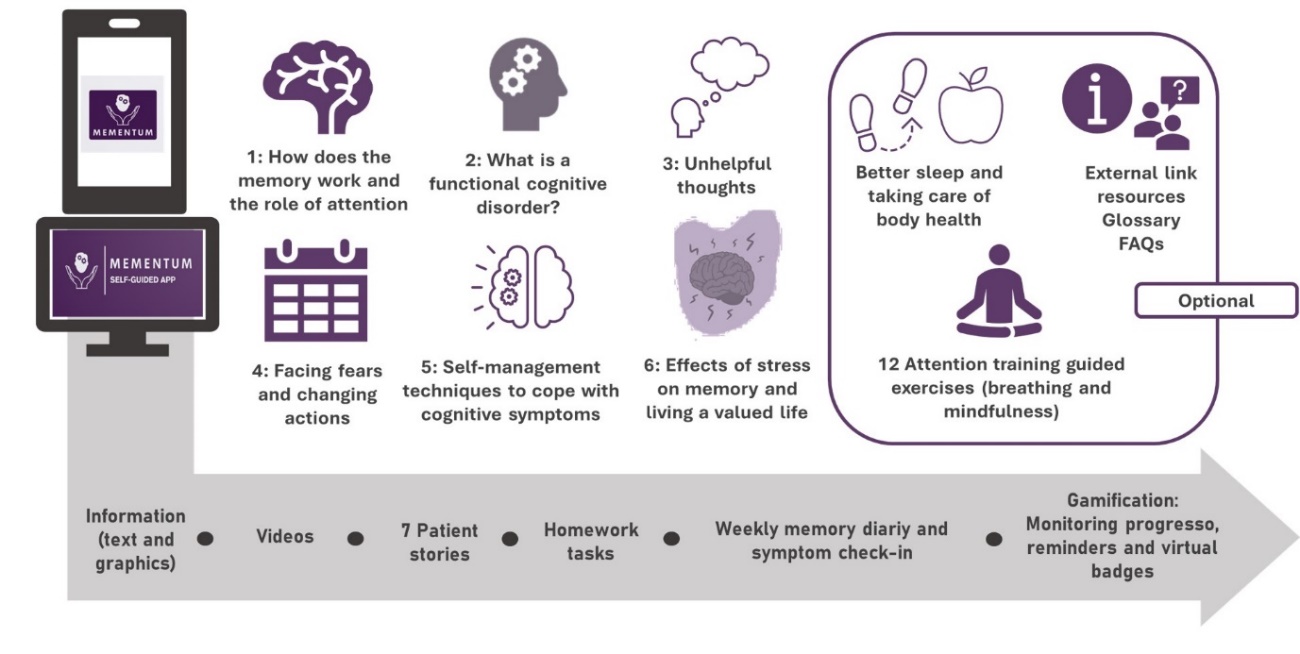


**Supplemental Figure 2.** Distribution of number of login days during the study (frequency represents the number of patients per each login days count).


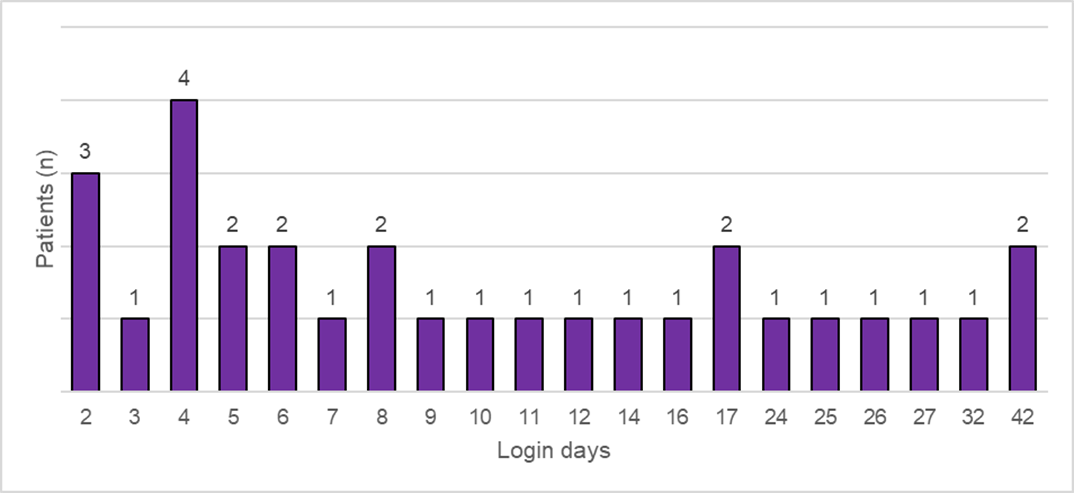


**Supplemental Figure 3.** Association between expected treatment benefit and perceived credibility prior to starting the intervention and clinical outcome change from baseline to follow-up, dividing participants by the 75th percentile of credibility/expectancy scores (Wilcoxon rank sum tests). QoL: quality-of-life measured by EQ-5D-5L VAS scale. WSAS: Work and Social Adjustment Scale. N=26, p>0.05 for all associations tested.


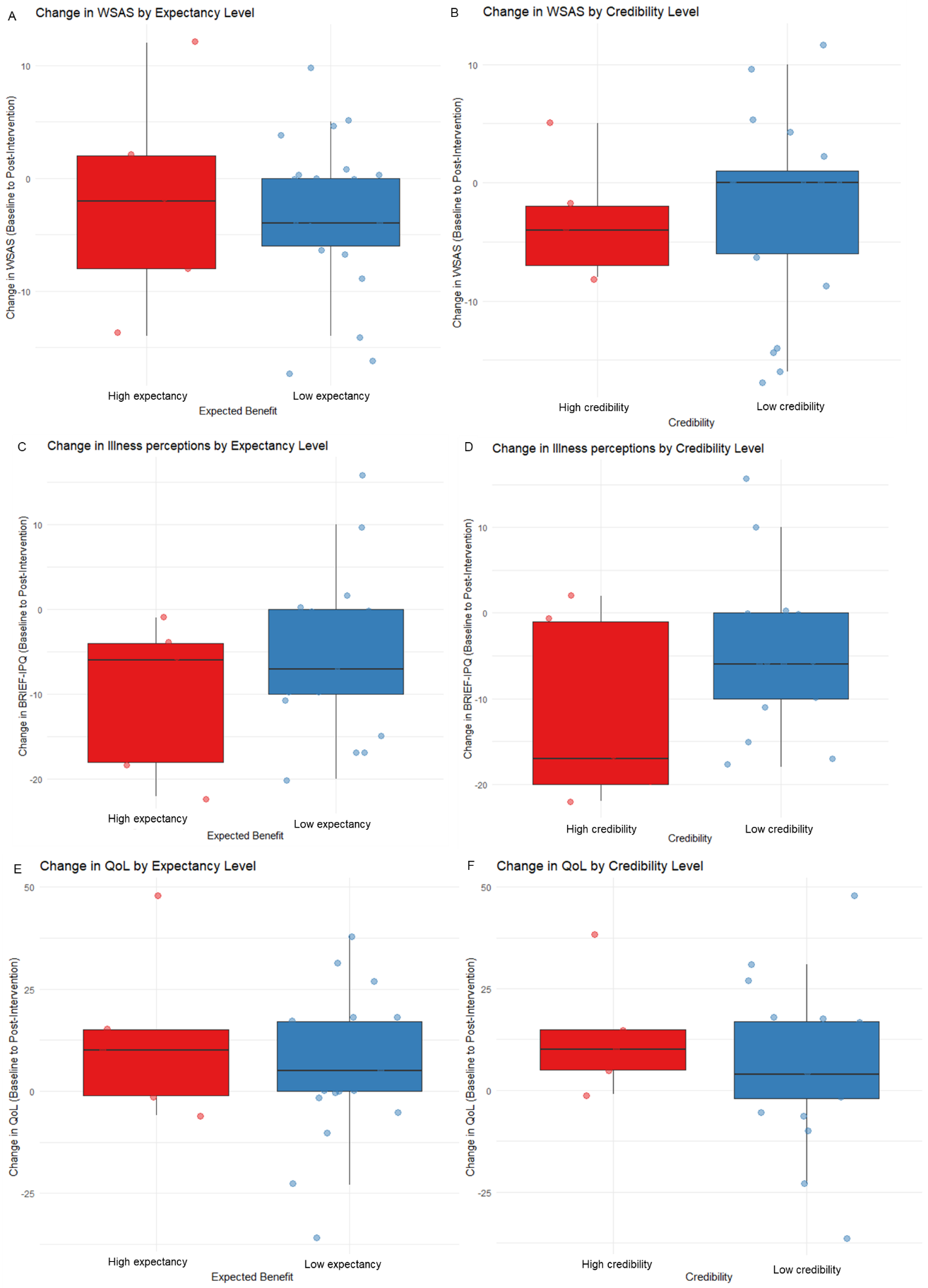


**Supplement Figure 4.** Entries in symptom check-in (total=103) among 15 possible symptoms (frequency/count for each symptom during the study period).


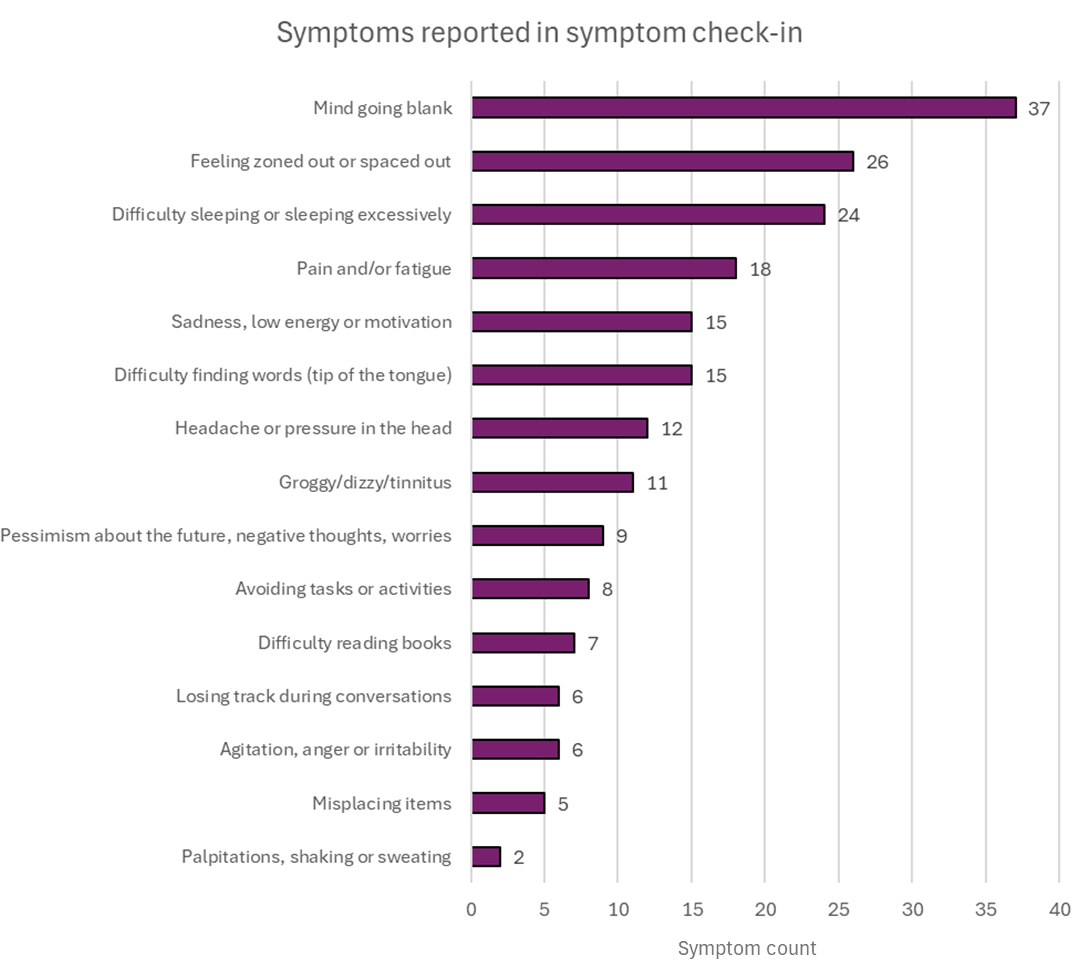


**Supplemental Figure 5.** Clinical global impression-improvement scale (A), subjective memory (5-point Likert scale) (B) and work and social functioning (C), at baseline and follow-up visits.


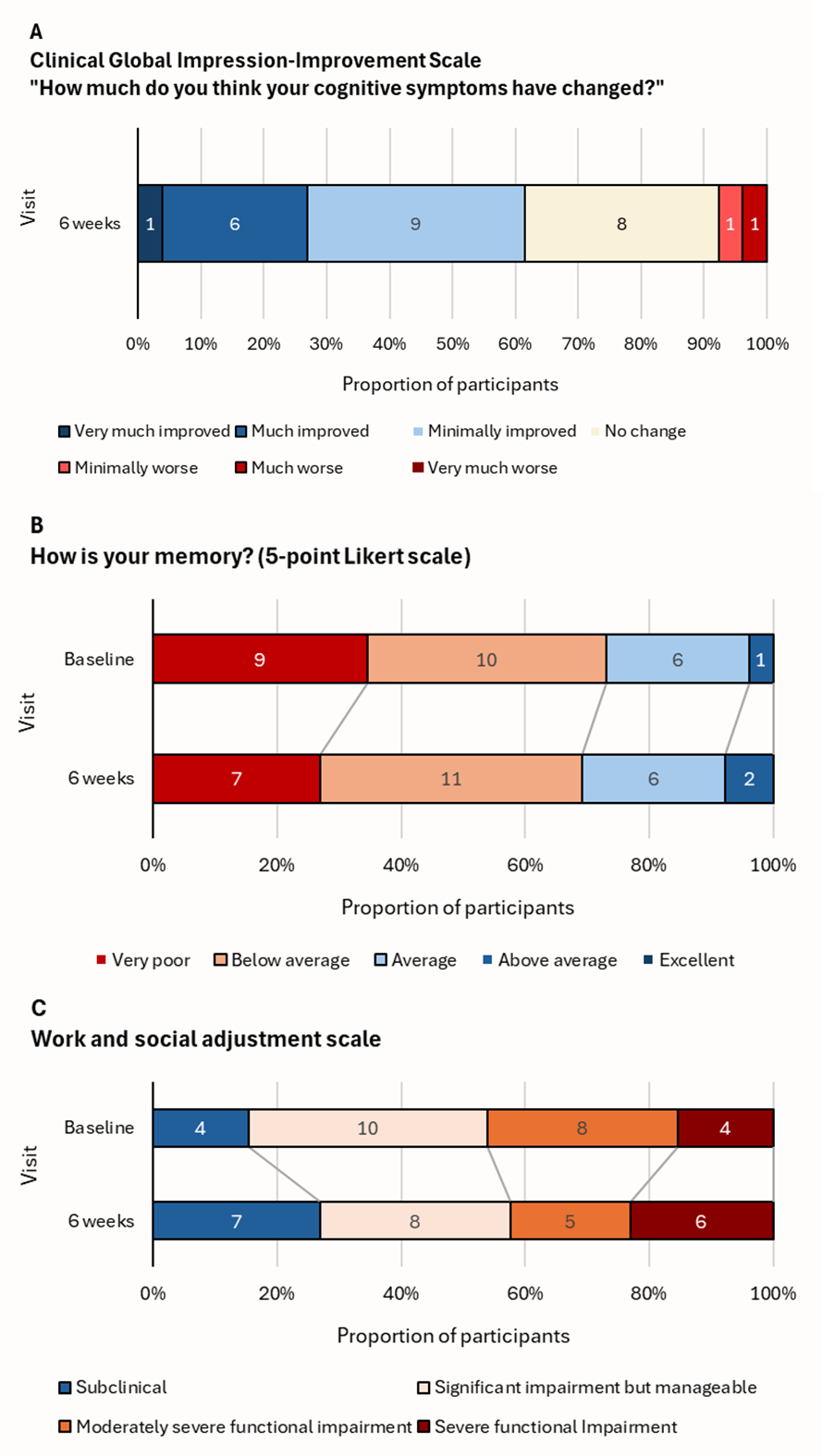


**Supplemental Figure 6.** EQ-5D-5L individual domains (A-mobility; B-self-care; C-usual activities; D-Pain/discomfort and E-anxiety/depression) at baseline and follow-up. Data are presented in frequency/count (N=26).


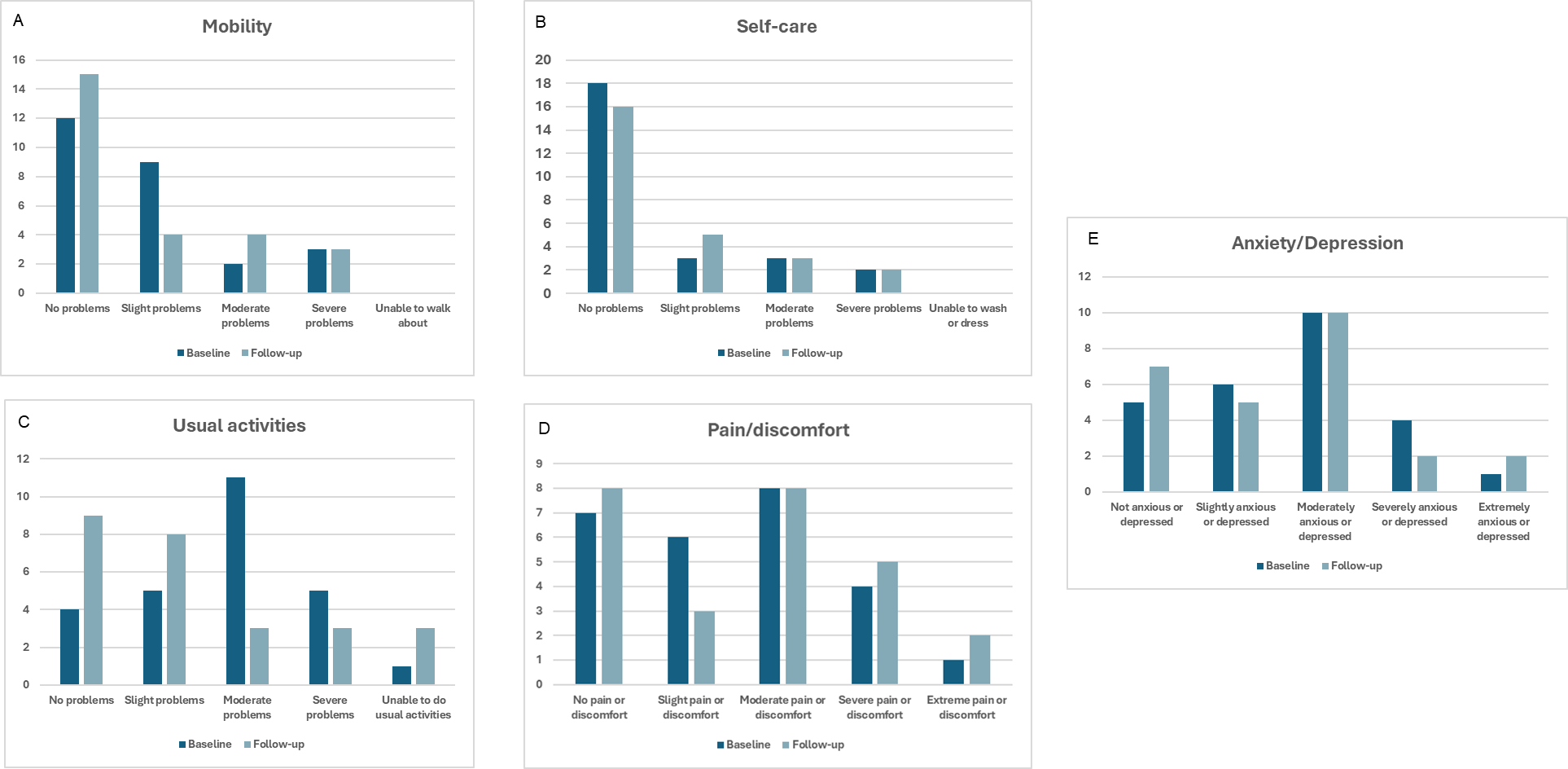


**References**

1. Cabreira V FL, Stone J, Carson, A. Development of a self-help digital intervention for functional cognitive disorder: a theory- and user-centred approach. JMIR Preprints (submitted) 2025.

2. McDonough IM, McDougall GJ, LaRocca M, Dalmida SG, Arheart KL. Refining the metamemory in adulthood questionnaire: a 20-item version of change and capacity designed for research and clinical settings. Aging & mental health. 2020;24(7):1054-63.

3. Broadbent E, Petrie KJ, Main J, Weinman J. The brief illness perception questionnaire. J Psychosom Res. 2006;60(6):631-7.

4. Gandy M, Heriseanu AI, Balakumar T, Karin E, Walker J, Hathway T, et al. The wellbeing neuro course: a randomised controlled trial of an internet-delivered transdiagnostic psychological intervention for adults with neurological disorders. Psychological Medicine. 2023:1-11.

5. Kroenke K, Spitzer RL, Williams JB. The PHQ-9: validity of a brief depression severity measure. Journal of general internal medicine. 2001;16(9):606-13.

6. Jordan P, Shedden-Mora MC, Löwe B. Psychometric analysis of the Generalized Anxiety Disorder scale (GAD-7) in primary care using modern item response theory. PLoS One. 2017;12(8):e0182162.

7. Spence M, Moss-Morris R, Chalder T. The Behavioural Responses to Illness Questionnaire (BRIQ): A new predictive measure of medically unexplained symptoms following acute infection. Psychological medicine. 2005;35:583-93.

8. Mundt JC, Marks IM, Shear MK, Greist JM. The Work and Social Adjustment Scale: a simple measure of impairment in functioning. The British Journal of Psychiatry. 2018;180(5):461-4.

9. Feng YS, Kohlmann T, Janssen MF, Buchholz I. Psychometric properties of the EQ-5D-5L: a systematic review of the literature. Quality of life research : an international journal of quality of life aspects of treatment, care and rehabilitation. 2021;30(3):647-73.

10. Busner J, Targum SD. The clinical global impressions scale: applying a research tool in clinical practice. Psychiatry (Edgmont (Pa : Township)). 2007;4(7):28-37.

11. Boß L, Lehr D, Reis D, Vis C, Berking M, Ebert D. Reliability and Validity of Assessing User Satisfaction With Web-Based Health Interventions. Journal of medical Internet research. 2016;18:e234.

12. Devilly G, Borkovec T. Psychometric properties of the Credibility/Expectancy Questionnaire. Journal of Behavior Therapy and Experimental Psychiatry. 2000;31:73-86.

13. Zhou L, Bao J, Setiawan IMA, Saptono A, Parmanto B. The mHealth App Usability Questionnaire (MAUQ): Development and Validation Study. JMIR mHealth and uHealth. 2019;7(4):e11500.

14. Rozental A, Kottorp A, Forsström D, Månsson K, Boettcher J, Andersson G, et al. The Negative Effects Questionnaire: psychometric properties of an instrument for assessing negative effects in psychological treatments. Behav Cogn Psychother. 2019;47(5):559-72.
